# Development of a tool for prediction of ovarian cancer in patients with adnexal masses: Value of plasma fibrinogen

**DOI:** 10.1371/journal.pone.0182383

**Published:** 2017-08-24

**Authors:** Veronika Seebacher, Stefanie Aust, David D’Andrea, Christoph Grimm, Elisabeth Reiser, Denise Tiringer, Hannah Von Mersi, Stephan Polterauer, Alexander Reinthaller, Samir Helmy-Bader

**Affiliations:** 1 Department for Gynecology and Gynecologic Oncology, Gynecologic Cancer Unit, Comprehensive Cancer Centre, Medical University of Vienna, Vienna, Austria; 2 Department of Urology, Medical University of Vienna, Vienna, Austria; 3 Karl Landsteiner Institute for General Gynecology and Experimental Gynecologic Oncology, Vienna, Austria; Suzhou University, CHINA

## Abstract

**Objective:**

To develop a tool for individualized risk estimation of presence of cancer in women with adnexal masses, and to assess the added value of plasma fibrinogen.

**Study design:**

We performed a retrospective analysis of a prospectively maintained database of 906 patients with adnexal masses who underwent cystectomy or oophorectomy. Uni- and multivariate logistic regression analyses including pre-operative plasma fibrinogen levels and established predictors were performed. A nomogram was generated to predict the probability of ovarian cancer. Internal validation with split-sample analysis was performed. Decision curve analysis (DCA) was then used to evaluate the clinical net benefit of the prediction model.

**Results:**

Ovarian cancer including borderline tumours was found in 241 (26.6%) patients. In multivariate analysis, elevated plasma fibrinogen, elevated CA-125, suspicion for malignancy on ultrasound, and postmenopausal status were associated with ovarian cancer and formed the basis for the nomogram. The overall predictive accuracy of the model, as measured by AUC, was 0.91 (95% CI 0.87–0.94). DCA revealed a net benefit for using this model for predicting ovarian cancer presence compared to a strategy of treat all or treat none.

**Conclusion:**

We confirmed the value of plasma fibrinogen as a strong predictor for ovarian cancer in a large cohort of patients with adnexal masses. We developed a highly accurate multivariable model to help in the clinical decision-making regarding the presence of ovarian cancer. This model provided net benefit for a wide range of threshold probabilities. External validation is needed before a recommendation for its use in routine practice can be given.

## Introduction

In western countries, epithelial ovarian cancer (EOC) is the second most common cancer of the female reproductive system and accountable for as many deaths as all other gynaecological cancers combined [1]. Early detection and treatment in high-volume centres by gynaecologic oncology surgeons is known to improve survival outcome [2, 3]. While adnexal masses are common sonographic findings in women of all ages, approximately 75% of tumours are found to be benign [4, 5]. Identifying those with a high risk of having ovarian cancer often poses a daily clinical challenge. Especially in young women, ovarian cancer is rare balancing the wish for preservation of fertility [6]. It is therefore of major importance to accurately differentiate between benign and malignant adnexal masses in order to avoid unnecessary surgical procedures and to deliver optimal care to those who are likely to harbour an ovarian cancer.

Prediction models have been generated to help with these risk-stratifications [7, 8]. However, none has received wide spread acceptance and use in routine clinical practice. Ultrasound features suggesting malignancy, such as ascites, increased vascularization, solid components, tumour size, papillary projections, and irregular cyst walls, are the basis for ultrasound based algorithms (e.g. the International Ovarian Tumour Analysis (IOTA) models) [4, 9–10]. Biomarkers, such as the tumour marker CA-125, have been validated and incorporated into such algorithms [6, 11–13]. Unfortunately, serum CA-125 levels are frequently normal in borderline ovarian tumours as well as early stage invasive ovarian cancer, and can be falsely elevated in benign conditions, particularly in premenopausal women [14–16]. To complement the effect of CA-125, models combining different biomarkers have been developed. The Risk of Ovarian Malignancy Algorithm (ROMA), incorporating measurements of CA-125 and the human epididymal secretory protein 4 (HE-4) performed particularly well in premenopausal women in a prospective, multicentre trial, leading to it’s approval by the U.S. Food and Drug Administration (FDA) [17]. However, other evaluations of the ROMA have reported conflicting results, doubting the beneficial effect of adding HE-4 to CA-125 [18]. Another biomarker-based diagnostic test, approved by the FDA, utilizes a five biomarker-combination and is available under the trade name OVA1 (Vermillion, Inc). Again, data on the test’s ability to outperform CA-125 measurements alone are conflicting [13, 19].

Fibrinogen is a key protein in the coagulation pathway and one of the major acute phase proteins, strongly linked to inflammation and stress. It has been found have prognostic value for various malignant tumours including ovarian cancer [20–27]. We have previously reported that pre-operative plasma fibrinogen is independently associated with ovarian cancer presence in patients with adnexal masses [25]. This study was limited by the failure to adjust for the effects of ultrasound results and its statistical design. For a biomarker to change clinical decision-making, it needs to improve the predictive accuracy for an event beyond that achieved by a multivariable analysis and add a net benefit in decision-analysis across a range of probabilities considered as relevant [28–30].

The aim of the present study was to validate these findings in a larger cohort, to find an optimal cut-off value for fibrinogen and to develop a nomogram as risk-prediction model for malignant ovarian tumours in patients with adnexal masses of unknown dignity.

## Materials and methods

### Patients and clinical management

Overall, 3,234 patients underwent surgery for suspected adnexal masses at a single tertiary care centre between 2000 and 2012. Of these, pre-operative plasma fibrinogen levels were available in 1,754 patients. After exclusion of patients with missing pre-operative CA-125 and on pre-operative ultrasound details, 906 patients remained eligible for inclusion into the present study. Patients with inflammatory processes, pregnancy related adnexal masses, and those with non-epithelial pathology on final histological result were previously excluded. Clinical and pathological data were retrospectively extracted from a prospectively maintained database. The study was approved by the institutional review board (Project # 1062/2015). The patient data was de-identified and handled in accordance with ethical standards of good scientific practice.

Patients were treated, as previously described [25], by laparoscopic or open ovarian cystectomy or salpingo-oophorectomy. In patients with EOC or borderline tumour of the ovary (BOT), surgery was extended to ensure adequate staging and, when necessary, complete resection of all visible tumour. If primary cytoreductive surgery was not feasible, patients with EOC were treated with neoadjuvant chemotherapy and interval debulking surgery.

Patients’ assessment prior to surgery included blood tests, a physical examination, a transvaginal ultrasound, and, in case of suspected malignancy, further imaging, such as computed tomography (CT) and / or magnetic resonance imaging (MRI). Postmenopausal status was defined as > 1 year of amenorrhoea or > 50 years of age in the case of previous hysterectomy. If criteria were present suspecting malignancy on ultrasound examination, as suggested by the individual examiner, ultrasound was classified as “presence of malignancy criteria (M-criteria) on ultrasound”. For CA-125 we used the established threshold of >35.0 U/mL [8].

### Fibrinogen measurements

Plasma fibrinogen levels were determined by the Clauss method [31] using clotting reagents. According to the manufacturer, the intra-assay variability was 3.5%. Plasma fibrinogen levels between 180 and 400 mg/dL were defined as normal by our laboratory. Yet, we aimed to determine an optimal cut-off for prediction of ovarian cancer within the cohort of the present study as described below.

### Statistics

Categorical variables are presented as numbers and proportions, continuous variables as medians (interquartile range [IQR]). Group differences in categorical and continuous variables were analysed using chi-square, Kruskal-Wallis and Mann-Whitney U tests. The optimal cut-off value for plasma fibrinogen was estimated by receiver operating characteristic (ROC) curve analysis. Potential predictors of ovarian cancer (OC, including both BOT and EOC) were analysed by uni- and multivariate logistic regression, including menopausal status, the presence of M-criteria on ultrasound, serum CA 125 levels > 35 U/mL, and elevated plasma fibrinogen according to the determined optimal cut-off value. Estimates are given as odd ratios (OR) and 95% confidence interval. The multivariate model combined all significant variables of the univariate analysis.

The regression coefficients of all significant variables in multivariate analysis were used to generate a nomogram for calculating the patient-specific probabilities of OC. A split-sample internal validation procedure was performed to establish that the model worked sufficiently among patients other than those whose data generated the model. Therefore, the entire cohort was randomly divided into two sub-cohorts with equal size and distribution of variables forming the test and the validation sample.

Values for each of the model’s covariates were mapped to points on a scale ranging from 0–10, with total points obtained for each model’s covariate mapped to the probability of OC associated with that combination of covariate values [32]. The predictive accuracy of the model was assessed by its discrimination and calibration. Discrimination was measured by the area under the receiver operating characteristic curve (AUC). The AUC measures the model’s ability to discriminate between patients with or without OC. An AUC of 0.5 indicates that the model provides no predictive discrimination, in other words, the model’s value would be like tossing a coin, while a value of 1.0 indicates perfect discrimination between patients with or without OC [33]. Calibration, which compares predicted with actual pathology result, was evaluated by calibration curves for both the test and the validation sample [32].

A decision curve analysis (DCA) was applied to explore the clinical value of our newly derived model by increasing the net benefit over a realistic range of threshold probabilities. In this study, the threshold probability represented the risk of a patient to be diagnosed with OC [28, 29].

All statistical tests were two sided, with significance set at a P-value <0.05. Analyses were conducted with SPSS 24.0 (SPSS 24.0.0, SPSS Inc., Chicago, IL, USA) and STATA 14.1 (Stata Corp. LP, College Station, TX, USA).

## Results

### Patients’ characteristics

The overall prevalence of OC in our cohort was 26.6% with 13.3% in pre- and 43.1% in postmenopausal patients. EOC was found in 190 patients (21%) and BOT in 51 (5.6%). Pathological results in patients with benign tumours were as follows: endometrioma: n = 154 (17.0%), teratoma: n = 103 (11.4%), simple benign cyst: n = 187 (20.6%), serous cystadenoma: n = 94 (10.4%), mucinous cystadenoma: n = 69 (7.6%), fibroma/Brenner tumour: n = 49 (5.4%), and other benign ovarian masses: n = 9 (1%). Pathological results in patients with OC were as follows: serous BOT: n = 29 (3.2%), mucinous BOT: n = 20 (2.2%), endometrioid BOT: n = 2 (0.2%), serous adenocarcinoma: n = 123 (13.6%), mucinous adenocarcinoma: n = 11 (1.2%), endometrioid adenocarcinoma: n = 35 (3.9%), clearcell carcinoma: n = 5 (0.6%), and undifferentiated carcinoma: n = 16 (1.7%). FIGO stages I, II, III, and IV were diagnosed in 40 (16.7%), 22 (9.1%), 120 (49.8%), and 20 (8.3%) patients, respectively. In 39 patients (16.2%), information on FIGO stage was missing. Pre-operative patients’ characteristics, ultrasound findings, CA-125 serum levels and plasma fibrinogen levels broken down by histological features are shown in Table 1.

**Table 1:** Pre-operative characteristics in 906 patients who underwent surgery for suspected adnexal masses.

	All	Benign	BOT	EOC	P value
	N = 906	N = 665 (73.4%)	N = 51 (5.6%)	N = 190 (21.0%)	
**Age,** median (IQR)	46 (35–61)	42 (32–55)	49 (37–60)	61 (50–70)	<0.001^1^
**Menopausal status**					<0.001^2^
Premenopausal (%)	502 (55.4)	435 (65.4)	27 (52.9)	40 (21.1)	
Postmenopausal (%)	404 (44.6)	230 (34.6)	24 (47.1)	150 (78.9)	
**Additional imaging (CT or MRI)**					<0.001^2^
Yes (%)	204 (22.5)	46 (6.9)	24 (47.1)	134 (65.7)	
No (%)	702 (77.5)	619 (93.1)	27 (52.9)	56 (29.5)	
**Ultrasound characteristics**					
*Maximal diameter (mm)*, median (IQR; NA: n = 39)	104 (71–149)	109.7 (67–138)	159 (89–210)	125 (90–180)	<0.001^1^
*Ascites*					<0.001^2^
Yes (%)	173 (19.1)	60 (9)	8 (15.7)	105 (55.3)	
No (%)	424 (46.8)	360 (54.1)	22 (43.1)	42 (22.1)	
NA	309 (34.1)	245 (36.8)	21 (41.2)	43 (22.6)	
*Localisation*					<0.001^2^
unilateral (%)	671 (74.1)	512 (77)	40 (78.4)	119 (62.6)	
bilateral (%)	235 (25.9)	153 (23)	11 (21.6)	71 (37.4)	
*M-Criteria present*					<0.001^2^
Yes (%)	355 (39.2)	179 (26.9)	32 (62.7)	144 (75.8)	
No (%)	551 (60.8)	486 (73.1)	19 (37.3)	46 (24.2)	
**CA125 (kU/l)** median (IQR)	27.2 (14–94.2)	20 (12.3–39)	28.6 (17–75.2)	485.5 (161.3–1310)	<0.001^1^
**CA125 > 35.0 kU/l**					<0.001^2^
Yes (%)	379 (41.8)	177 (26.6)	21 (41.2)	181 (95.3)	
No (%)	527 (58.2)	488 (73.4)	30 (58.8)	9 (4.7)	
**Fibrinogen (mg/dl)** median (IQR)	344 (286–427.3)	329 (275–386)	349 (297–412)	473.5 (366.5–580.3)	<0.001^1^
**Fibrinogen > 342 mg/dl**					<0.001^2^
Yes (%)	465 (51.3)	227 (41.7)	29 (56.9)	159 (83.7)	
No (%)	441 (48.7)	388 (58.3)	22 (43.1)	31 (16.3)	

^1^Kruskal-Wallis test.

^2^Chi-square test.

IQR = interquartile range; BOT = borderline tumour of the ovary; EOC = epithelial ovarian cancer; CT = computer tomography; MRI = magnetic resonance imaging.

### Fibrinogen as a predictive marker for ovarian cancer

Sensitivity and specificity for the detection of OC using our laboratory’s upper limit of normal for plasma fibrinogen of 400 mg/dl were 55.2% and 78.5%, respectively. However, as the normal range does not necessarily reflect on the association between fibrinogen and malignant growth, we aimed to determine an optimal cut-off value for fibrinogen for the detection of ovarian malignancy in patients with adnexal masses. ROC curve analysis revealed an AUC of 0.74 (95% confidence interval [CI] 0.7–0.78) for fibrinogen to detect OC. In comparison, the ROC curve analyses for CA-125, the presence of M-criteria on ultrasound, and menopausal status revealed AUCs of 0.88 (95% CI 0.85–0.9), 0.73 (95% CI 0.69–0.77), and 0.69 (95% CI 0.65–0.73) for detecting OC, respectively. As missing an ovarian malignant tumour would have serious fatal consequences, we aimed to keep the false negative rate low thereby maximizing sensitivity. In accordance to the utility-based decision theory [34], based on ROC curve analysis, we chose a level of 342 mg/dl plasma fibrinogen as optimal cut-off value, reaching a sensitivity, specificity, positive predictive value (PPV), and negative predictive value (NPV) for detecting OC of 78.0%, 58.3%, 40.4%, and 87.9% respectively. This cut-off value was used for further analyses, as it reflected the most optimal balance between NPV and PPV for OC. In comparison, sensitivity, specificity, PPV, and NPV for CA 125 serum level > 35 kU/L were 83.8%, 73.3%, 53.3%, and 92.6%, and for the presence of M-criteria on ultrasound 73.0%, 73.1%, 49.6%, and 88.2%, respectively.

In addition, we wanted to assess the added effect of elevated fibrinogen (>342 mg/dl) to elevated CA-125 (>35 kU/L) to detect ovarian malignancy in our cohort. A Spearman’s correlation was run to assess the relationship between serum CA-125 and fibrinogen levels. There was only a weak correlation between the two biomarkers, which was statistically significant (Spearman’s Rho (*r*_*s*_*) = 0*.*299*; *p<0*.*001*). Thus, this indicates that only about 8.9% of the variance of one of the above biomarkers was explained by the other. The correlation between serum CA-125 and fibrinogen levels is shown in the scatter plot of S1 Fig. Next, we compared distributions of ovarian malignancy and benign ovarian tumours between the groups with both markers low, with only one of either fibrinogen or CA-125 elevated, or with both biomarkers elevated. In 22 (9.1%) out of 241 patients with ovarian malignancy only fibrinogen was elevated while CA-125 was found to be within normal range. Distributions of OC within the above named groups and the respective NPV, PPV, and number needed to treat (NNT) to detect one ovarian malignancy are shown in Table 2.

**Table 2:** Diagnostic performance of serum fibrinogen and CA-125 to detect ovarian malignancy in 906 patients with adnexal masses.

	Benign N = 665	Ovarian malignancy N = 241	PPV	NPV	NNT
**CA-125 and fibrinogen low (%)**	286 (43)	17 (7.1)	-	-	-
**Fibrinogen elevated only^a^ (%)**	202 (30.4)	22 (9.1)	40.4%^c^	58.3%^c^	3.5^c^
**CA-125 elevated only^b^ (%)**	102 (15.3)	36 (14.9)	53.3%^d^	73.3a%^d^	2.2^d^
**CA-125 and fibrinogen elevated (%)**	75 (11.3)	166 (68.9)	68.9%^e^	88.7%^e^	1.7^e^

PPV = positive predictive value; NPV = negative predictive value; NNT = numbers needed to treat to detect one ovarian malignancy.

^a^fibrinogen >342 mg/dl.

^b^CA-125 >35 kU/L.

^c^diagnostic performance of a test including only serum fibrinogen levels.

^d^diagnostic performance of test including only serum CA-125 levels.

^e^diagnostic performance of a test including both serum CA-125 and fibrinogen levels.

### Risk-predicting model and decision curve analysis

**Fig 1:**
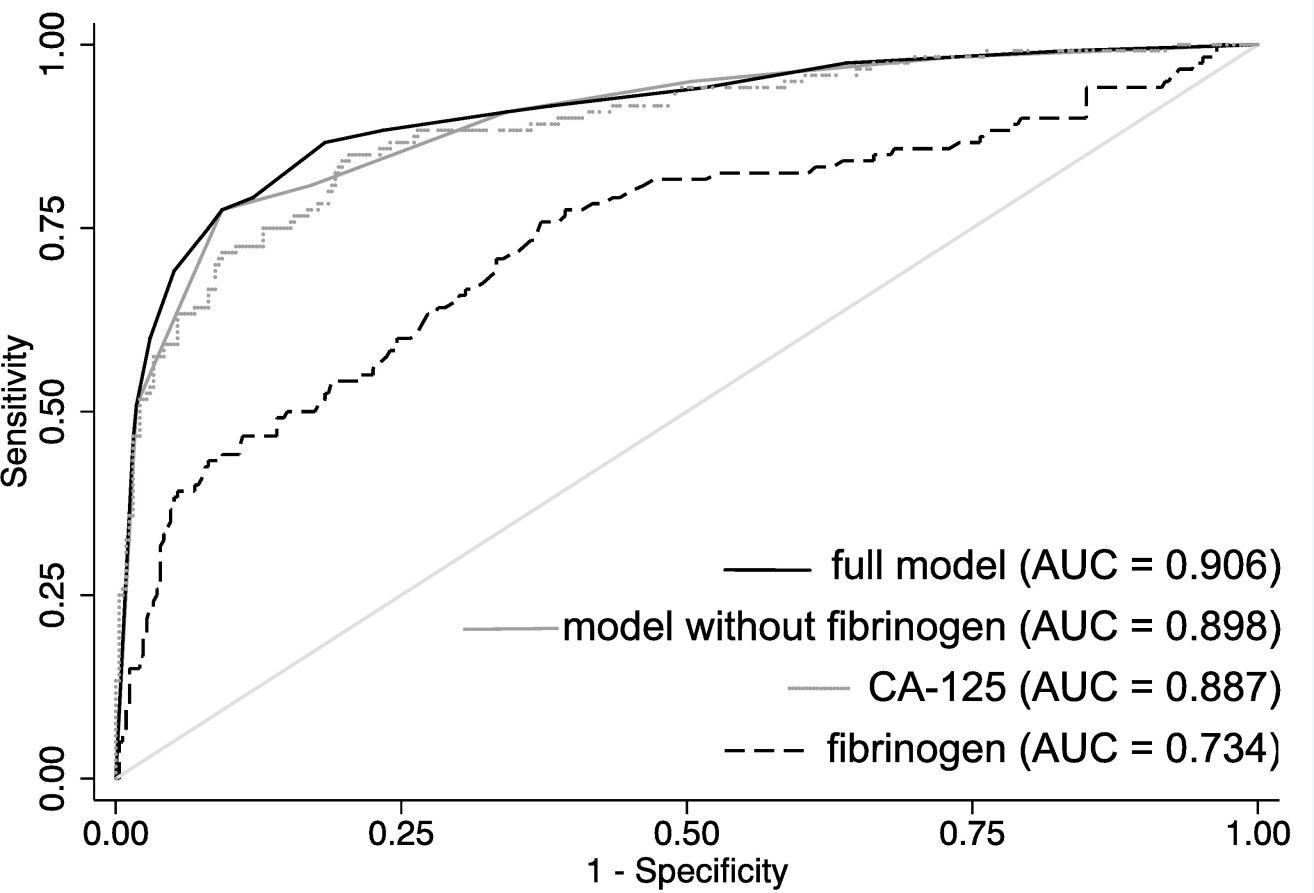
ROC-curves representing the accuracy for detection of ovarian malignancy in patients with adnexal masses for CA-125, fibrinogen, the full model (including fibrinogen, CA-125, malignancy-criteria on ultrasound), and the model without fibrinogen. AUC = area under the curve.

In both uni- and multivariate logistic regression analyses, plasma fibrinogen > 342 mg/dl, CA-125 > 35 kU/L, postmenopausal status (or age > 50 years), and the presence of M-criteria on ultrasound were independently associated with a higher risk for the presence of OC. These four variables formed the basis for the nomogram. Results of uni- and multivariate logistic regression analyses are shown in Table 3. The differential benefit of adding fibrinogen to a base model that includes CA-125, menopausal status, and evaluation of M-criteria on ultrasound was approximately one per-cent. This would translate into 1 in 100 patients receiving more accurate prediction of ovarian cancer helping them to tailor treatment. The different ROC curves for fibrinogen, CA-125, the model with, and the model without the addition of fibrinogen are shown in Fig 1.

**Table 3:** Predictive factors for ovarian malignancy in uni- and multivariate logistic regression analyses of 906 patients who underwent surgical removal of their adnexal masses.

	Univariate analysis		Multivariate analysis	
	OR (95% CI)	*P* value	OR (95% CI)	*P* value
**Postmenopausal status or age > 50 years**	4.91 (3.55–6.79)	<0.001	3.88 (2.53–5.96)	<0.001
**Presence of M-criteria on ultrasound**	7.35 (5.27–10.25)	<0.001	6.11 (4.01–9.31)	<0.001
**CA-125 > 35 kU/l**	14.28 (9.73–20.95)	<0.001	13.2 (8.46–20.59)	<0.001
**Fibrinogen > 342 mg/dl**	4.96 (3.53–6.99)	<0.001	3.52 (2.26–5.48)	<0.001

OR = Odds Ratio; CI = Confidence Interval.

**Fig 2:**
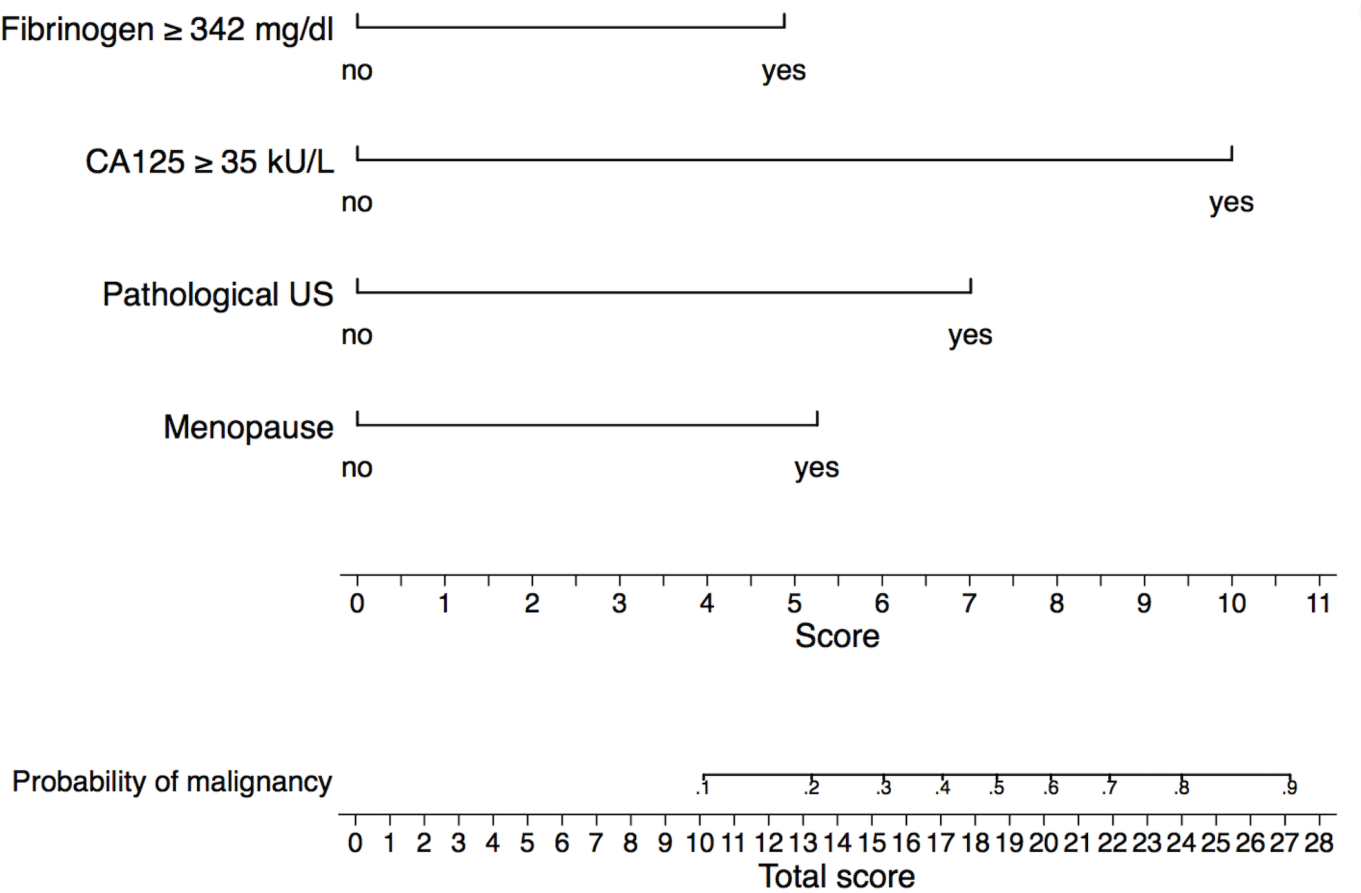
Nomogram to predict ovarian cancer in patients with adnexal masses. To use the nomogram, locate the patient’s variable on the corresponding axis; draw a line to the “score” axis, sum the scores, and draw a line from the “total score” axis to the “probability of malignancy” axis.

**Fig 3:**
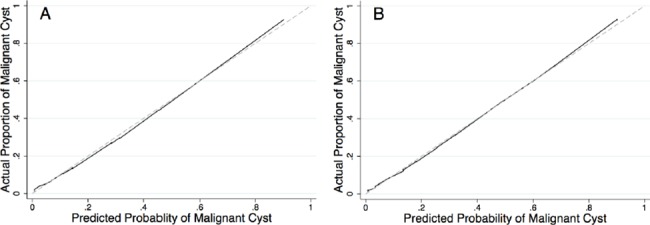
**Calibration plots for assessing the performance of the nomogram to predict ovarian cancer in patients with adnexal masses in (A) the test sample (n = 453) and (B) the validation sample (n = 453).** In both (A) and (B), the dashed line represents the ideal reference line where the predicted probabilities estimated from the model would match the observed proportion of patients with ovarian cancer.

Fig 2 shows the nomogram for predicting OC in patients with adnexal masses based on menopausal status, presence of M-criteria on ultrasound, elevated CA-125 (> 35 U/ml), and elevated plasma fibrinogen (> 342 g/dl). Test and validation samples were well matched on all the characteristics investigated (S1 Table). The overall predictive accuracy of the model, as measured by AUC, was 0.91 (95% CI 0.87–0.94) and 0.90 (95% CI 0.87–0.94) for the test and the validation samples, respectively. The calibration plots shown in Fig 3 compare observed (vertical axis) toward predicted OC (horizontal axis) for both the test and the validation samples. Pearson test did not reveal significant differences between the predicted and the observed probabilities for OC for both the test and the validation samples (*p = 0*.*84* and *p = 0*.*35*, respectively), meaning that the nomogram was well calibrated.

The nomogram is used by locating the score assigned for each predictive factor as depicted on the topmost “score” scale. Then a sum is calculated across all patient characteristics to obtain the “total score” that is eventually converted into the desired probability of OC. Of note, a higher total score corresponds with a higher probability of OC. For instance, we picture a premenopausal patient with an adnexal mass, presence of M-criteria on ultrasound, CA-125 of 60 kU/L, and plasma fibrinogen level of 300 mg/dl. By locating each of the characteristics on the corresponding scale and drawing a vertical straight line down to the “score” scale, we obtain scores of around 0, 7, 10, and 0, respectively, adding up to a total score of 17. Locating this value on the “total score” scale and drawing a line to the “probability of malignancy” scale a probability of 40% for OC can be estimated. On the other hand, if this same patient had a plasma fibrinogen level of 400 mg/dl, this would add 5 points to the score of 17, leading to a total score of 23. The estimated probability for OC would thereby change to 70%.

**Fig 4:**
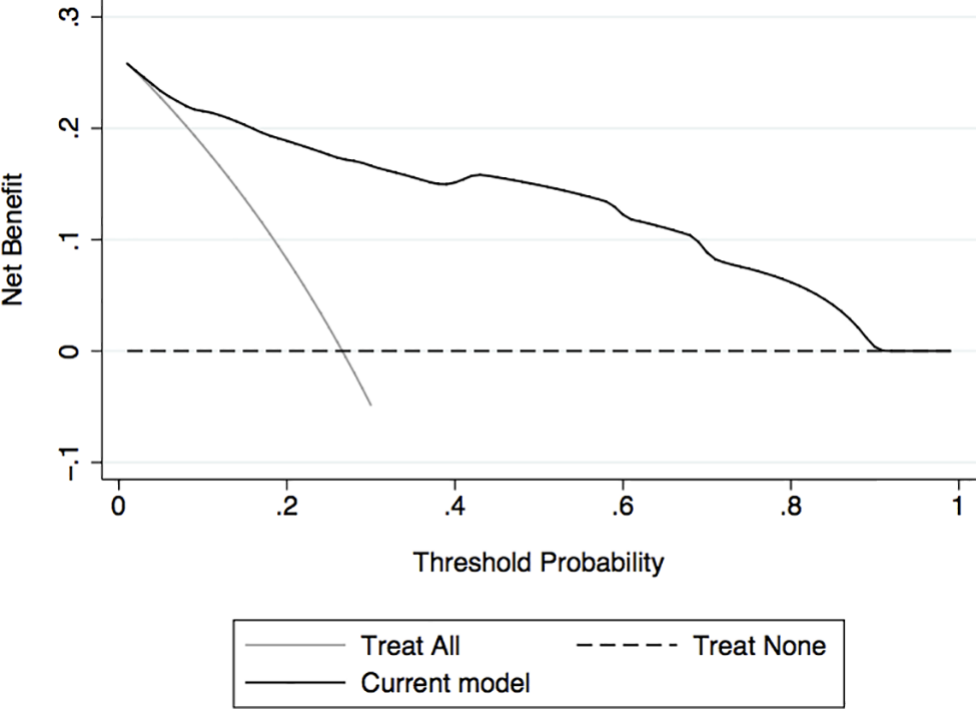
Decision curve analysis of the effect of the presented nomogram for predicting ovarian cancer in patients with adnexal masses. Assumption was made that the identification of ovarian cancer would lead to surgery. Net benefit of the presented nomogram is plotted against threshold probabilities for ovarian cancer compared with the strategies of treating all patients with adnexal masses or no one. The nomogram showed an improved net benefit between 5% and 90% threshold probability.

In the DCA (Fig 4), our model provided net benefit in predicting OC throughout nearly the entire range of threshold probabilities compared to treating all patients with surgery, or alternatively, treating no one. Only below a threshold of 5% probability for OC there would be no difference between applying our model and treating all patients. On the other hand, above a threshold of 90% probability for OC, deciding for treatment based on our model would have the same net benefit compared to treating no one. Net benefits and the percentages of interventions avoided for each respective threshold are listed in Table 4. For example, if a clinician would decide for surgery in patients with adnexal masses starting at a threshold probability for OC of 20%, by applying the present model the net reduction of unnecessary surgeries compared to treating all patients would be about 42.5 per 100 patients. In other words, at this probability threshold, deciding for surgery on the basis of our model is equivalent to a strategy that reduced the rate of surgery by 42.5% in patients without OC, without missing any OC. Moreover, if we perform surgery based on our prediction model, compared to treating none, the net consequence is equivalent to remove true-positive OC in 19 patients per 100 and treating no unaffected patients. As shown in Table 4, the net benefit of our model increases with cumulative threshold probabilities.

**Table 4:** Net benefits for the nomogram to predict ovarian cancer in patients with adnexal masses. Assumption was made that the identification of ovarian cancer would lead to surgery.

Threshold probability for ovarian cancer, %	Net benefit in treating all	Net benefit for the nomogram	Interventions avoided, %	Increase in net benefit
10	.18	.22	28.3	.03
20	.08	.19	42.5	.11
40	-.22	.15	56.1	.37
60	-.83	.12	63.6	.95
80	-2.7	.06	68.3	2.73

## Discussion

Adnexal masses are commonly found in women of all ages and the decision to surgically explore them or not often poses a clinical challenge. Surgical intervention carries inherent risks as well as the possibility of removing an ovary. Accurate prediction of the risk of harbouring an OC could help avoiding overtreatment as well as it could facilitate timely referral for surgical intervention in those at high risk for ovarian malignancy.

We confirmed the value of pre-operative plasma fibrinogen as a strong predictor for OC in a large cohort of patients with adnexal masses. Pre-operative fibrinogen was associated with the presence of OC in uni- and multivariate analyses that adjusted for the effects of menopausal status, ultrasound-based suspicion for malignancy, and elevated plasma CA-125. Based on these factors, we built a highly accurate nomogram (AUC 0.91) to help guide clinicians to tailor treatment in women who present with an adnexal mass. To assess whether this tool is worth using, we performed a DCA. The curve generated by DCA revealed, that over-treatment could be reduced by the use of the proposed nomogram, compared to treating all or no patients for a wide range of threshold probabilities.

Apart from being one of the key proteins within the formation of blood clots and inflammatory processes, plasma fibrinogen levels have been found to be elevated in patients with a poor prognosis in various malignant tumours including OC [20–27]. Moreover, our study group has previously investigated the predictive role of plasma fibrinogen to distinguish between benign and malignant ovarian tumours [25]. The results of the present study confirm the independent predictive value of elevated plasma fibrinogen to be associated with an increased risk for OC in patients with suspected adnexal masses in a very large cohort of patients. Indeed, as sole predictive marker for OC, plasma fibrinogen proved more accurate than the presence of criteria suspecting malignancy on ultrasound (M-criteria) or a postmenopausal status, while less accurate than plasma CA-125. The ROC curves for fibrinogen to detect OC were similar in both the present and the previously published study (AUC 0.74 vs. AUC 0.78, respectively). The slight variation between the AUC values of the current and the previous study is probably due to the now larger patient sample. In addition, we established a new cut-off value for plasma fibrinogen by ROC curve analysis with the aim of maximizing sensitivity while keeping it balanced with an acceptable specificity.

In contrast to our previous logistic regression model [25], where we included patients’ age, serum CA-125 (< vs. ≥ 35 kU/L) and plasma fibrinogen (with the laboratories normal range as cut-off), we used our newly determined cut-off value for fibrinogen, replaced patients’ age by menopausal status, and added the presence of M-criteria on ultrasound to the present model. Elevated plasma fibrinogen levels remained independently associated with an increased risk for OC in patients with adnexal masses even after adjusting for the effects of these established risk factors.

Various scoring systems have been investigated to pre-operatively estimate the risk for malignancy in patients with adnexal masses, two of which have been approved by the FDA. The ROMA incorporates measurements of CA-125 and HE4 along with the menopausal status of the patient. The score was originally evaluated in a prospective, multicentre, blinded clinical trial in 471 patients with adnexal masses [17]. The results of this study demonstrated a highly favourable sensitivity and specificity to detect OC of 93.8% and 74.9%, respectively. Since then, several studies have been performed reporting partly divergent results. A prospective study in 389 patients with adnexal masses, for example, reported no benefit of ROMA in comparison to the use of CA-125 alone for the detection of ovarian malignancy (ROC-AUC_ROMA_ = 0.898 vs. ROC-AUC_CA-125_ = 0.877) [18]. The variance between the studies has been ascribed to differences in the composition of the patient cohorts [35]. The second test approved by the FDA, OVA1^TM^ Ovarian Triage Test, combines a panel of five biomarkers for ovarian cancer (CA-125, transthyretin, apolipoprotein A1, ß2-microglobulin, and transferrin) identified through serum proteomics using SELDI-TOF-MS. This multivariate index assay was demonstrated to perform better than CA-125 in detecting OC in a clinical trial in 524 patients diagnosed with adnexal mass, 161 of which were found to have ovarian malignancy. The OVA1 test thereby provided a sensitivity and specificity of 93% and 43%, respectively, with a PPV of 42% and a NPV of 93% [13]. However, in another study evaluating sera from 1.069 patients included in the Prostate Lung Colon and Ovary (PLCO) Cancer Screening Trial, the OVA1 test was not found to improve the detection of ovarian malignancy compared to CA-125 measurements alone [19]. Compared to the results of the ROMA and OVA1 test, the accuracy of our model was relatively similar. Obviously, comparing the results of our study to those of prospective clinical trials is lacking validity. Yet, a possible advantage of our model is the measurement of serum fibrinogen, which is readily available and cheap to perform, while costs for the FDA-approved tests are estimated between USD 60–130 for the ROMA and USD 600–650 for the OVA1 test [35].

The authors of a recent meta-analysis, evaluating numerous scoring systems for adnexal masses, summarized their findings by strongly recommending the incorporation of ultrasound-based prediction models into pre-operative characterization of adnexal pathology [7]. The IOTA study group has done substantial work by developing and validating diagnostic models for adnexal masses based on standardised ultrasound examination protocols and definitions [9, 10, 36–39]. A meta-analysis, comparing various ultrasound-based prediction models for OC, suggested the IOTA group’s models Logistic Regression (LR) 2 and Simple Rules (SR) to have the strongest test performance [40]. While transvaginal ultrasound is the key tool used in daily clinical practice for evaluating adnexal masses, a general criticism of the use of ultrasound as diagnostic test for distinguishing between a benign and malignant ovarian tumour is, that it is subjective and its performance strongly depends upon the experience and skills of the respective examiner. Due to lack of training and / or shortage of time, risk assessment in daily clinical practice is often based only on a subjective evaluation of ultrasound and, eventually, on pre-operative CA-125 measurement.

A nomogram is a prediction tool that incorporates various risk factors with the attempt to quantify the individualized probability of an outcome using a continuous risk scale. The graphic depiction of the probability of a particular outcome on a continuous scale, which is usually 0–100%, thereby provides a user-friendly interface, which does not require computer software for interpretation [30]. The model evaluated within the present study revealed a strong test performance with an AUC of 0.91. The simplicity of its design and the few numbers of variables make it easy to use in daily clinical practice. As CA-125 was the strongest predictive marker in our collective we wanted to assess the added value of fibrinogen. By performing a Spearman correlation we could demonstrate that the two biomarkers had only a weak correlation. This would emphasize a potential benefit of combining the two biomarkers, as they do not seem to be only each other’s surrogate parameter. Furthermore, we compared distributions of OC between groups of patients generated according to the finding of elevated serum levels of fibrinogen alone, CA-125 alone, none, or both. We found that approximately 9% of patients with OC had elevated serum fibrinogen levels without elevation of CA-125. Based on the findings of serological biomarkers only, in these patients OC would have possibly been missed. Consequently, the PPV and NPV for detection of OC based on CA-125 and fibrinogen only were highest if both biomarkers were elevated. To assess the added value of fibrinogen to the model in a whole (including CA-125, fibrinogen, M-criteria on ultrasound, and menopausal status), we compared the ROC curves of the model with and without the addition of fibrinogen. The addition of fibrinogen to the model led to an increase of accuracy to detect OC of approximately one per-cent. This might seem to be only a minor improvement. Yet, fibrinogen is a cheap serological marker that is routinely measured in most patients prior to a surgical intervention. Therefore, improving the accuracy of prediction of ovarian malignancy in one out of a hundred patients that present with adnexal masses is justifying the addition of fibrinogen to the model.

While the AUC is indicating that our nomogram provides a strong predictive accuracy, it does not incorporate information on consequences and, therefore, cannot tell us whether the model is worth using at all. To examine the potential clinical impact of our predictive model, we performed DCA, a statistical method to estimate the clinical consequences of using predictive models [28, 29]. The curve estimated by DCA for our nomogram demonstrated, that the use of our model to predict OC in patients with adnexal masses, and consequently, to help guiding clinicians and patients in the decision making process of whether to perform surgery or not, provided a net benefit relative to the two strategies of treating all, or alternatively, no one. The net benefit was given for threshold probabilities of OC between 5% and 90% and increased directly proportionally to the increment of threshold probabilities. The key advantage of DCA is the option of varying the threshold probability over an appropriate range. This is of importance, as patients may reasonably disagree about the appropriate threshold for deciding for surgery or even oophorectomy for an adnexal mass, depending on their age, their wish to preserve fertility and their general health status.

Strengths of the present study include the single institution uniform approach to care and its relatively large sample size, which enabled us to implement a split-sampling approach for validation of statistical results. However, there are some limitations that deserve to be mentioned. Within this study, data of a prospectively maintained database were retrospectively analysed. Therefore, the study has short-comings characteristic for a retrospective design, such as patient selection and incomplete data acquisition. Moreover, the model was based on patients who were selected for surgery only. Therefore, we cannot be certain, that the test performance of our model would remain unchanged if applied in a cohort of patients, of whom some would opt for expectant management. All patients were referred to and treated at a tertiary care centre specialized in gynaecologic oncology. Hence, the patient cohort of the present study might not be representative for the general population of women diagnosed with an adnexal mass at a clinic of a local gynaecologist. In addition, due to the retrospective design and the relatively long study period, ultrasound criteria used to determine suspicion for malignancy within this study were not standardized. However, in all patients, ultrasound was done by highly experienced examiners at our institution, a tertiary referral centre for suspicious adnexal masses, and can therefore be regarded as sound.

In conclusion, the current clinical management of patients with adnexal masses, detected by ultrasound results in a high rate of unnecessary surgical interventions [41].

Therefore, more accurate tools are needed to pre-operatively estimate the risk of OC in these patients. The nomogram described in the present study may guide decision-making process towards the most adapted surgical treatment options or expectant management strategies. DCA of the nomogram shows clinical benefit both for circumstances asking for a more hesitant attitude and for those allowing a more liberal approach towards surgical treatment of an adnexal mass. However, in order to prove generalizability, the current model requires external multi-institutional cohort validation. Further studies evaluating such predictive tools are needed especially to elucidate the benefit of combining our model with standardised ultrasound-based scores.

## Supporting information

S1 Fig Scatter plot representing the correlation between serum CA-125 and serum fibrinogen levels in 906 patients with adnexal mass. (TIF)

S1 Table Pre-operative characteristics in 906 patients treated with surgery for adnexal masses comparing test (n = 453) and validation samples (n = 453). (DOCX)

## References

[1] Siegel R. Naishadham D, Jemal A. Cancer statistics 2013. CA Cancer J Clin 2013;63:11–30. doi: 10.3322/caac.21166 23335087 10.3322/caac.21166

[2] Engelen MJ, Kos HE, Willemse PHB, Aalders JG, de Vries EG, Schaapveld M, et al Surgery by consultant gynecologic oncologists improves survival in patients with ovarian carcinoma. Cancer 2006;106:589–98. doi: 10.1002/cncr.21616 16369985 10.1002/cncr.21616

[3] Woo YL, Kyrgiou M, Bryant A, Everett T, Dickinson H. Centralization of services for gynecological cancer–a Chochrane systematic review. Gynecol Oncol 2012; 126:286–90. doi: 10.1016/j.ygyno.2012.04.012 22507534 10.1016/j.ygyno.2012.04.012

[4] Alcázar JL, Castillo G, Jurado M, García GL. Is expectant management of sonographically benign adnexal cysts an option in selected asymptomatic premenopausal women? Hum Reprod 2005;20:3231–4. doi: 10.1093/humrep/dei206 16024535 10.1093/humrep/dei206

[5] Bristow RE, Smith A, Zhang Z, Chang DW, Crutcher G, Fung ET, et al Ovarian malignancy risk stratification of the adnexal mass using a multivariate index assay. Gynecol Oncol 2013;128:252–9. doi: 10.1016/j.ygyno.2012.11.022 23178277 10.1016/j.ygyno.2012.11.022

[6] Dearking A, Aletti G, McGree M, Weaver AL, Sommerfield MK, Cliby WA. How relevant are ACOG and SGO guidelines for referral of adnexal mass? Obstet Gynecol 2007;110:841–8. doi: 10.1097/01.AOG.0000267198.25223.bc 17906018 10.1097/01.AOG.0000267198.25223.bc

[7] Dodge JE, Covens AL, Lacchetti C, Elit LM, Le T, Devries-Aboud M, et al and the Gynecologic Cancer Disease Site Group. Preoperative identification of a suspicious adnexal mass: a systematic review and meta-analysis. Gynecol Oncol 2012;126:157–66. doi: 10.1016/j.ygyno.2012.03.048 22484399 10.1016/j.ygyno.2012.03.048

[8] Timmerman D, Testa AC, Bourne T, Ferrazzi E, Ameye L, Konstantinovic ML, et al Logistic regression model to distinguish between the benign and malignant adnexal mass before surgery: a multicenter study by the International Ovarian Tumor Analysis Group. J Clin Oncol 2005;23:8794–801. doi: 10.1200/JCO.2005.01.7632 16314639 10.1200/JCO.2005.01.7632

[9] Timmerman D, Testa AC, Bourne T, Ameye L, Jurkovic D, Van Holsbeke C, et al Simple ultrasound-based rules fort he diagnosis of ovarian cancer. Ultrasound Obstet Gynecol 2008;31:681–90. doi: 10.1002/uog.5365 18504770 10.1002/uog.5365

[10] Timmerman D, Ameye L, Fischerova D, Epstein E, Melis GB, Guerriero S, et al Simple ultrasound rules to distinguish between benign and malignant adnexal masses before surgery: prospective validation by IOTA group. BMJ 2010;341:c6839 doi: 10.1136/bmj.c6839 21156740 10.1136/bmj.c6839 PMC3001703

[11] Bast RC Jr, Skates S, Lokshin A, Moore RG. Differential diagnosis of a pelvic mass: improved algorithms and novel biomarkers. Int J Gynecol Cancer 2012;22:S5–S8. doi: 10.1097/IGC.0b013e318251c97d 22543921 10.1097/IGC.0b013e318251c97d PMC3389992

[12] Kaijser J, Van Gorp T, Sayasneh A, Vergote I, Bourne T, Van Calster B, et al Differentiating stage I epithelial ovarian cancer from benign disease in women with adnexal tumors using biomarkers or the ROMA algorithm. Gynecol Oncol 2013;130:398–9. doi: 10.1016/j.ygyno.2013.04.472 23656998 10.1016/j.ygyno.2013.04.472

[13] Ueland FR, DeSimone CP, Seamon LG, Miller RA, Goodrich S, Podzielinski I, et al Effectiveness of a multivariate index assay in the preoperative assessment of ovarian tumors. Obstet Gynecol 2011;117:1289–97. doi: 10.1097/AOG.0b013e31821b5118 21606739 10.1097/AOG.0b013e31821b5118

[14] Engelen MJ, de Bruijn HW, Hollema H, ten Hoor KA, Willemse PH, Aalders JG, et al Serum CA 125, carinoembryonic antigen, and CA 19–9 as tumor markers in borderline ovarian tumors. Gynecol Oncol 2000;78:16–20. doi: 10.1006/gyno.2000.5811 10873403 10.1006/gyno.2000.5811

[15] Gotlieb WH, Soriano D, Achiron R, Zalel Y, Davidson B, Kopolovic J, et al CA 125 measurements and ultrasonography in borderline tumors of the ovary. Am J Obstet Gynecol 2000;83:541–6. 10.1067/mob.2000.105940 10992171

[16] Sevinc A, Adli M, Kalender ME, Camci C. Benign causes of increased serum CA-125 concentration. Lancet Oncol 2007;8:1054–5. doi: 10.1016/S1470-2045(07)70357-1 18054877 10.1016/S1470-2045(07)70357-1

[17] Moore RG, Miller MC, Disilvestro P, Landrum LM, Gajewski W, Ball JJ, et al Evaluation of the diagnostic accuracy of the risk of ovarian malignancy algorithm in women with a pelvic mass. Obstet Gynecol 2011;118:220–8. 10.1097/AOG.0b013e318224fce2 PMC3594110 21775843

[18] Van Gorp T, Cadron I, Despierre E, Daemen A, Leunen K, Amant F, et al HE4 and CA125 as a diagnostic test in ovarian cancer: prospective validation oft he Risk of Ovarian Malignancy Algorithm. Br J Cancer 2011;104:863–70. doi: 10.1038/sj.bjc.6606092 21304524 10.1038/sj.bjc.6606092 PMC3048204

[19] Moore LE, Pfeiffer RM, Zhang Z, Lu KH, Fung ET, Bast RC Jr. Proteomic biomarkers in combination with CA 125 for detection of epithelial ovarian cancer using prediagnostic serum samples from the Prostate, Lung, Colorectal, and Ovarian (PLCO) Cancer Screening Trial. Cancer 2012;118:91–100. doi: 10.1002/cncr.26241 21717433 10.1002/cncr.26241 PMC3385508

[20] Polterauer S, Grimm C, Seebacher V, Concin N, Marth C, Tomovski C, et al Plasma fibrinogen levels and prognosis in patients with ovarian cancer: a multicenter study. The Oncologist 2009a;14:979–85. doi: 10.1634/theoncologist.2009-0079 19776095 10.1634/theoncologist.2009-0079

[21] Polterauer S, Seebacher V, Hefler-Frischmuth K, Grimm C, Heinze G, Tempfer C, et al Fibrinogen plasma levels are an independent prognostic parameter in patients with cervical cancer. Am J Obstet Gynecol 2009b;6:647.e1-7. 10.1016/j.ajog.2009.01.008 19306966

[22] Polterauer S, Seebacher V, Hefler-Frischmuth K, Grimm C, Heinze G, Tempfer C, et al Fibrinogen plasma levels are an independent prognostic parameter in patients with cervical cancer. Am J Obstet Gynecol 2009b;6:647.e1-7. 10.1016/j.ajog.2009.01.008 19306966

[23] Seebacher V, Polterauer S, Grimm C, Husslein H, Leipold H, Hefler-Frischmuth K, et al The prognostic value of plasma fibrinogen levels in patients with endometrial cancer: a multi-centre trial. Br J Cancer 2010;102:952–6. doi: 10.1038/sj.bjc.6605547 20160724 10.1038/sj.bjc.6605547 PMC2844023

[24] Qui J, Yu Y, Fu Y, Feng Y, Xie X, Lu W. Preoperative plasma fibrinogen, platelet count and prognosis in epithelial ovarian cancer. J Obstet Gynaecol Res 2012;38:651–7. doi: 10.1111/j.1447-0756.2011.01780.x 22413879 10.1111/j.1447-0756.2011.01780.x

[25] Hefler-Frischmuth K, Lafleur J, Hefler L, Polterauer S, Seebacher V, Reinthaller A, et al Plasma fibrinogen levels in patients with benign and malignant ovarian tumors. Gynecol Oncol 2015;136:567–70. doi: 10.1016/j.ygyno.2014.12.041 25576886 10.1016/j.ygyno.2014.12.041

[26] Feng Z, Wen H, Bi R, Duan Y, Yang W, Wu X. Thrombocytosis and hyperfibrinogenemia are predictive factors of clinical outcomes in high-grade serous ovarian cancer patients. BMC Cancer 2016;16:43 doi: 10.1186/s12885-016-2070-2 26817451 10.1186/s12885-016-2070-2 PMC4730624

[27] Ma C, Lu B, Diao C, Zhao K, Wang X, Ma B, et al Preoperative neutrophil-lymphocyte ratio and fibrinogen level in patients distinguish between muscle-invasive bladder cancer and non-muscle-invasive bladder cancer. Onco Targets Ther 2016;9:4917–22. doi: 10.2147/OTT.S107445 27540305 10.2147/OTT.S107445 PMC4982501

[28] Vickers AJ, Elkin EB. Decision curve analysis: a novel method for evaluating prediction models. Med Decis Making 2006;26:565–74. doi: 10.1177/0272989X06295361 17099194 10.1177/0272989X06295361 PMC2577036

[29] Vickers AJ, Cronin AM, Elkin EB, Gonen M. Extensions to decision curve analysis, a novel method for evaluating diagnostic tests, prediction models and molecular markers. BMC Med Inform Decis Mak 2008;8:53 doi: 10.1186/1472-6947-8-53 19036144 10.1186/1472-6947-8-53 PMC2611975

[30] Shariat FS, Umberto C, Jeldres C, Karakiewicz PI. Can nomograms be superior to other prediction tools? BJUI 2008;103:492–7. 10.1111/j.1464-410X.2008.08073.x 18990135

[31] Clauss A. Rapid physiological coagulation method in determination of fibrinogen. Acta Haematol 1957;17:237–46 13434757 10.1159/000205234

[32] Harrell FE Jr, Lee KL, Pollock BG. Regression models in clinical studies: determining relationships between predictors and response. J Natl Cancer Inst 1988;80:1198–202. 3047407 10.1093/jnci/80.15.1198

[33] Hanley JA, McNeil BJ. The meaning and use of the area under a receiver operating characteristic (ROC) curve. Radiology 1982;143:29–36. doi: 10.1148/radiology.143.1.7063747 7063747 10.1148/radiology.143.1.7063747

[34] Greiner M, Pfeiffer D, Smith RD. Principles and practical application of the receiver operating characteristic analysis for diagnostic test. Prev Vet Med 2000;45:23–41. 10802332 10.1016/s0167-5877(00)00115-x

[35] Nolen BM and Lokshin AE. Biomarker testing for ovarian cancer: clinical utility of multiplex assays. Mol Diagn Ther 2013;17:139–46. doi: 10.1007/s40291-013-0027-6 23552992 10.1007/s40291-013-0027-6 PMC3670781

[36] Jacobs I, Oram D, Fairbanks J, Turner J, Frost C, Grudzinskas JG. A risk of malignancy index incorporating CA 125, ultrasound and menopausal status for the accurate preoperative diagnosis of ovarian cancer. Br J Obstet Gynaecol 1990;97:922–9. 2223684 10.1111/j.1471-0528.1990.tb02448.x

[37] Tingulstad S, Hagen B, Skjeldestad FE, Onsrud M, Kiserud T, Halvorsen T, et al Evaluation of a risk of malignancy index based on serum CA-125, ultrasound findings and menopausal status in the pre-operative diagnosis of pelvic masses. Br J Obstet Gynaecol 1996;103:826–31. 8760716 10.1111/j.1471-0528.1996.tb09882.x

[38] Timmerman D, Verrelst H, Bourne TH, De Moor B, Collins WP, Vergote I, et al Artificial neural network models fort he preoperative discrimination between malignant and benign adnexal masses. Ultrasound Obstet Gynecol 1999;13:17–25. doi: 10.1046/j.1469-0705.1999.13010017.x 10201082 10.1046/j.1469-0705.1999.13010017.x

[39] Van Calster B, Van Hoorde K, Valentin L, Testa AC, Fischerova D, Van Holsbeke C, et al International Ovarian Tumour Analysis (IOTA) group. Evaluating the risk of ovarian cancer before surgery using the ADNEX model to differentiate between benign, borderline, early and advanced stage invasive and secondary metastatic tumours: prospective multicentre diagnostic study. BMJ 2014;349:g5920 doi: 10.1136/bmj.g5920 25320247 10.1136/bmj.g5920 PMC4198550

[40] Kaijser J, Sayasneh A, Van Hoorde K, Ghaem-Maghami S, Bourne T, Timmerman D, et al Presurgical diagnosis of adnexal tumours using mathematical models and scoring systems: a systematic review and meta-analysis. Hum Reprod Update 2014;20:449–62. doi: 10.1093/humupd/dmt059 24327552 10.1093/humupd/dmt059

[41] Buys SS, Partridge E, Greene MH, Prorok PC, Reding D, Riley TL, et al PLCO Project Team. Ovarian cancer screening in the Prostate, Lung, Colorectal and Ovarian (PLCO) cancer screening trial: findings from the initial screen of a randomized trial. Am J Obstet Gynecol 2005;193:1630 doi: 10.1016/j.ajog.2005.05.005 16260202 10.1016/j.ajog.2005.05.005

