# Supplementary figures and images for: Development of a tool for prediction of ovarian cancer in patients with adnexal masses: Value of plasma fibrinogen
Source: PLoS One. 2017 Aug 24;12(8):e0182383. doi: 10.1371/journal.pone.0182383 (PMC5570374; doi:10.1371/journal.pone.0182383)

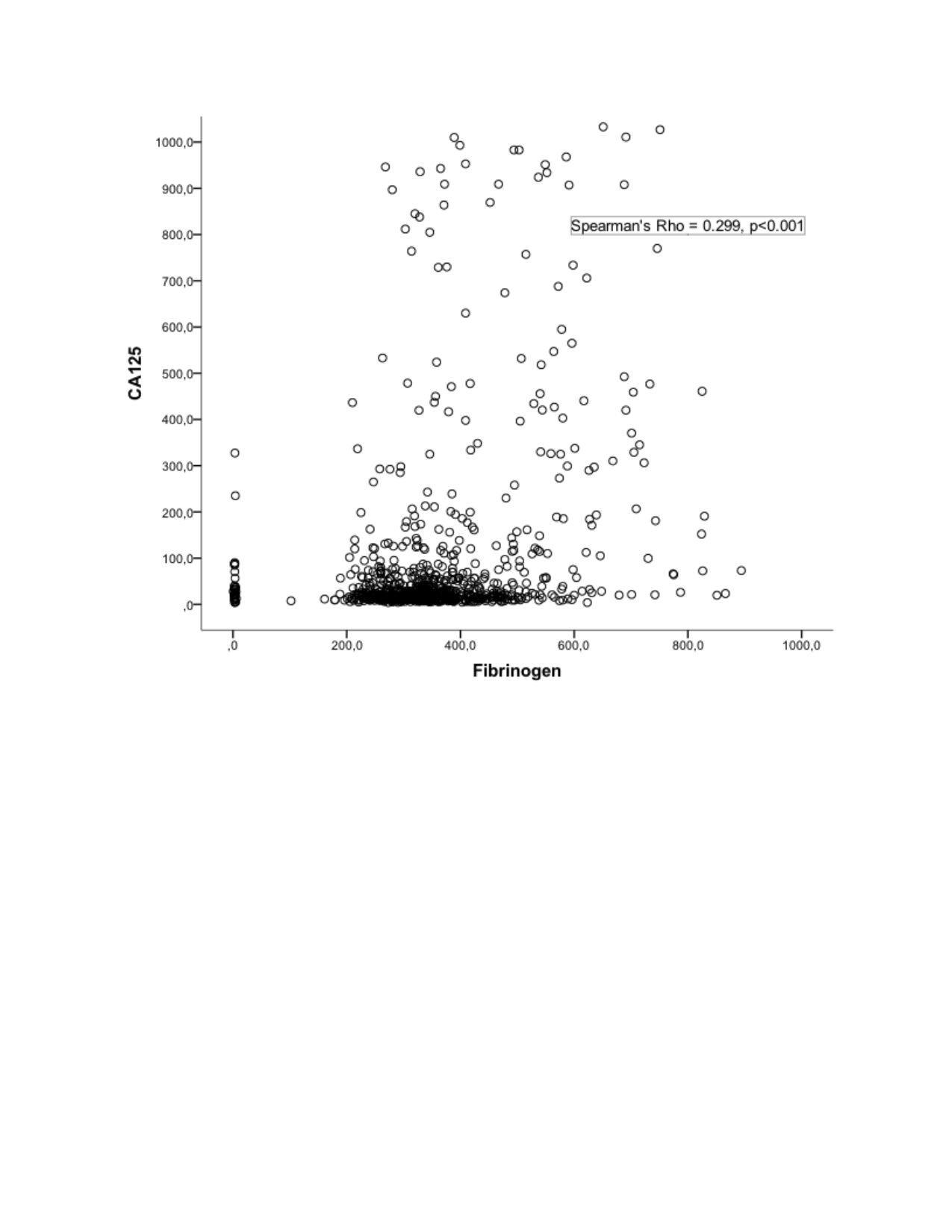

Supplement: S1 Fig — (TIF) [file pone.0182383.s001.tif]
