# Supplementary material for: Development of a tool for prediction of ovarian cancer in patients with adnexal masses: Value of plasma fibrinogen
Source: PLoS One. 2017 Aug 24;12(8):e0182383. doi: 10.1371/journal.pone.0182383 (PMC5570374; doi:10.1371/journal.pone.0182383)
Supplement: S1 Table — (DOCX) [file pone.0182383.s002.docx]

|  | Overall | Test sample | Validation sample | *p-value* |
| --- | --- | --- | --- | --- |
| Patients, n (%) | 906 (100) | 453 (50) | 453 (50) |  |
| Age, median (IQR) | 46 (35 – 61) | 46 (37 – 61) | 47 (34 – 60) | 0.2^1^ |
| Postmenopausal status, n (%) | 404 (44.6) | 203 (44.8) | 201 (44.4) | 0.9^2^ |
| Presence of M-criteria on ultrasound, n (%) | 355 (39.2) | 188 (41.5) | 167 (36.9) | 0.1^2^ |
| CA 125 ≥ 35 kU/L, n (%) | 379 (41.8) | 193 (42.6) | 186 (41.1) | 0.6^2^ |
| Fibrinogen ≥ 342 mg/dl, n (%) | 465 (51.3) | 229 (50.5) | 236 (52.1) | 0.6^2^ |

^1^Mann-Whitney U test; ^2^Chi-square test; IQR=inter-quartile range
